# Rubella Immunity in Pregnant Iranian Women: A Systematic Review and Meta-Analysis 

**DOI:** 10.22074/ijfs.2019.5562

**Published:** 2019-07-14

**Authors:** Milad Azami, Zahra Jaafari, Ali Soleymani, Gholamreza Badfar, Shamsi Abbasalizadeh

**Affiliations:** 1 Faculty of Medicine, Ilam University of Medical Sciences, Ilam, Iran; 2 Women’s Reproductive Health Research Center, Tabriz University of Medical Sciences, Tabriz, Iran; 3 HIV/STI Surveillance Research Center, WHO Collaborating Center for HIV Surveillance, Institute for Futures Studies in Health, Kerman University of Medical Sciences, Kerman, Iran; 4 Dezful University of Medical Sciences, Dezful, Iran; 5 Department of Pediatrics, Behbahan Faculty of Medical Sciences, Behbahan, Iran

**Keywords:** Immunity, Iran, Meta-Analysis, Pregnant Women, Rubella

## Abstract

Rubella infection within the first trimester of pregnancy may lead to adverse pregnancy outcomes. The present study was conducted to evaluate the immunity against rubella among the pregnant Iranian women. The steps of meta-analyses were conducted based on the MOOSE protocol and results were reported according to the PRISMA guideline. To review the associated English and Persian literature, a comprehensive search was conducted among the international databases such as Scopus, PubMed/Medline, Science Direct, Embase, Cochrane library, Web of Science and Google Scholar search engine as well as Iranian databases, until April 1, 2018 using the following medical subject headings (MeSH) keywords: ‘Pregnant’, ‘Gestational’, ‘Prenatal care’, ‘Complications of pregnancy’, ‘Pregnancy’, ‘Rubella infection’, ‘Prevalence, ‘Epidemiology’, ‘Immunity’, ‘Immunization’, ‘Antibody’, ‘Immunogenicity’ and ‘Iran’. Cochran’s Q test and I2 index were used to investigate heterogeneity in the studies. Random effects model was used to estimate the rate of rubella immunity. The obtained data were analyzed using Comprehensive Meta-Analysis Ver.2. Fifteen studies constituting 7,601 pregnant Iranian women met the inclusion criteria. The overall pooled rubella immunity rate was 90.1% [95% confidence interval (CI): 86.1-93.1]. Rubella immunity rates were respectively 88.6% (95% CI: 80.6-93.6) and 91.5% (95% CI: 88.1-93.9) before and after national vaccine program. Rubella immunity rates were 91.4% (95% CI: 87.8-94.0) and 87.2% (95% CI: 74.3-94.1) based on the enzyme-linked immunosorbent assay (ELISA) and haemagglutination-inhibition (HAI) methods, respectively. There was no significant association between rubella immunity and vaccination program (P=0.398), diagnostic methods (P=0.355), geographic regions (P=0.286), quality of the studies (P=0.751), occupation (P=0.639), residence (P=0.801), and year of the studies (P=0.164), but it was significantly associated with age (P<0.001).

Despite high rubella immunity among the pregnant Iranian women, anti-rubella antibody screening is recommended for all women of childbearing age.

## Introduction

Rubella virus is an important pathogen worldwide and a member of the genus Rubivirus in the Togaviridae family. This human virus is transmitted through aerosols and usually causes benign infections in children and young adults (1, 2). Rubella virus in adults may also cause severe inflammation and joint pain (3). Moreover, this infection may cause premature birth, low birth weight (4), miscarriage, stillbirth (5) and congenital rubella syndrome (CRS) during the first trimester of pregnancy (4-6). This syndrome is characterized by fetal abnormalities, including mental retardation, blindness, deafness (7), heart defects, cataracts (6), hepatomegaly and jaundice (8). Rubella infection is dangerous during pregnancy, especially during the first trimester. The rate of congenital malformations in newborns is 50, 25 and 17% for the first, second and third months, respectively (9-11).

Currently, there is no antiviral treatment for rubella (2), but an efficient vaccine is available against rubella (2, 3). The World Health Organization (WHO) recommends a comprehensive strategy for rubella and CRS control and eventual elimination in conjunction with rubella elimination, using measles/rubella or measles/mumps/rubella vaccines (12).

According to studies conducted in different regions of the world, the immunity against rubella has been reported to be diverse from 66-100% (13-16). Many studies have been conducted in Iran and these studies have reported the rubella immunity rate of 75-96% in pregnant women (17-20). Given the importance of this subject, the need for a comprehensive study is necessary. The analysis includes study of rubella immunity in pregnant Iranian women before and after introduction of the vaccine and assessing influence factors on sero-status

A more clear picture of the problem dimensions in the community can be provided through systematic review of all documentation and combining them with meta-analysis (21-23). This study was conducted to assess rubella immunity in pregnant Iranian women.

## Materials and Methods

### Study protocol

To identify relevant studies, a systematic review was performed on cross-sectional and case-control studies related to rubella immunity in pregnant women. The review was carried out in accordance with Meta-analysis of observational studies in epidemiology (MOOSE) protocol and results were reported according to the Preferred Reporting Items for Systematic Reviews and Meta-Analyses (PRISMA) guideline (23). To avoid bias in the study, search, selection of studies, quality assessment and data extraction were independently performed by two researchers. In case of discrepancies in the result of the two researchers, the study was referred to the third researcher.

### Search strategy

To evaluate related English and Persian literatures, a comprehensive search was conducted in six national databases including: Iranian Research Institute for Information Science and Technology (IranDoc; https://irandoc. ac.ir), Scientific Information Database (SID; http://www. sid.ir/), Barakat Knowledge Network System (http:// health.barakatkns.com), Iranian National Library (http:// www.nlai.ir/) and Regional Information Center for Science and Technology (RICST; http://en.ricest.ac.ir/), Magiran (http://www.magiran.com/) and six international databases including: Scopus, PubMed/Medline, Science Direct, Embase, Cochrane Library, Web of Science (ISI) and Google Scholar search engine. The search was done without time limit until April 1, 2018. High-sensitivity search was independently carried out by two researchers who were familiar with searching in databases (Azami M. and Jaafari Z.). Medical Subject Headings (MeSH) keywords were ‘Pregnant’, ‘Gestational’, ‘Pregnancy’, ‘Rubella infection’, ‘Prevalence, ‘Epidemiology’, ‘Immunity’, ‘Immunization’, ‘Antibody’, ‘Prenatal care’, ‘Immunogenicity’ and ‘Iran’. The combined search was performed using Boolean operators (AND and OR). Combined search in PubMed database is shown as follows: (“Pregnant”[Title/ Abstract] OR “Pregnancy”[Title/Abstract] OR“Prenatal care” [Title/Abstract] OR “Gestational”[Title/Abstract]) AND (“Rubella”[Title/Abstract] OR “Immunity”[Title/ Abstract] OR “Immunogenicity”[Title/Abstract] OR “Immunization”[Title/Abstract] OR “Antibody”[Title/ Abstract] OR “Prevalence”[Title/Abstract] OR “Epidemiology”[Title/Abstract]) AND (“Iran”[Title/Abstract/ Affiliation]).

After the end of search, the title of collected articles was entered into EndNote™ resource management to find similar articles. Manual search was also carried out by reviewing the reference list of relevant articles.

### Inclusion and exclusion criteria

The inclusion criteria according to PICO (Evidence Based Medicine) (24) were: i. **P**opulation: pregnant Iranian women, ii. **I**ntervention: serological tests such as enzyme-linked immunosorbent assay (ELISA) or haemagglutination-inhibitory (HAI) methods to confirm immunity against rubella, iii. **C**omparison: it can show the immunity seroprevalence in terms of age, occupation and place of residence, and iv. **O**utcome: estimating the overall seroprevalence of rubella immunity in pregnant women and other risk factors.

Exclusion criteria were: i. Non-random sample for seroprevalence of rubella immunity, ii. Non-pregnant women, iii. Non-Iranian sample, iv. Low-quality studies, v. Duplicate studies, and vi. Review articles, case reports and letters to the editor.

### Methodological quality assessment

The researchers evaluated quality of the selected studies using a scoring system, according to the modified Newcastle Ottawa Scale (NOS) for cross-sectional studies (25). The attainable minimum score was five and the articles that received a minimum score underwent quality assessment and Metadata extraction processes.

### Data extraction

Data extraction form included the author’s name, age (mean ± SD), place of residence, sample size, study design, rubella immunity, before/after national vaccination program, diagnostic method, quality score and the number of event and total in case and control groups or odds ratio (OR) and 95% confidence interval (CI) for risk factors. The extracted data was compared by two researchers and shared with the third researcher in case of discrepancies and finally a consensus was reached to re-examine and compare the results. Specific questions or relevant ambiguities in the articles were asked from the author via email.

### Statistical analysis

The binomial distribution was used to estimate the standard error of rubella immunity in each study. OR index was calculated to evaluate the effect of age, occupation and place of residence on rubella immunity. Cochran’s Q test and I2 index were used to investigate heterogeneity in the studies. Interpretation in this regard was as follows: 0-24% indicates low heterogeneity, 25-49% indicates moderate heterogeneity, 50-75% indicates substantial heterogeneity and over 75% indicates high heterogeneity. To estimate the seroprevalence of rubella immunity and to measure the effect of age on rubella immunity rate due to high heterogeneity between studies, random-effects model was used. To measure the effect of occupation and place of residence on rubella immunity rate, due to low heterogeneity, the fixed effects model was used to combine data (26). We also conducted sensitivity analysis by removing one study at the same time to assess the stability of the meta-analysis results. Sub-group analysis and meta-regression of the rubella immunity were used to find the potential sources of heterogeneity. Sub-group analysis were divided based on five regions of Iran, national rubella vaccination program, diagnostic methods and quality of studies. Funnel plot and Egger and Begg’s tests were used to examine the publication bias. Finally, data were analyzed using Comprehensive Meta-Analysis software Ver.2 (Biostat, Inc. Company, U.S. and U.K.). The significance level was set at 0.05.

## Results

### Searching results and characteristics

**Fig 1:**
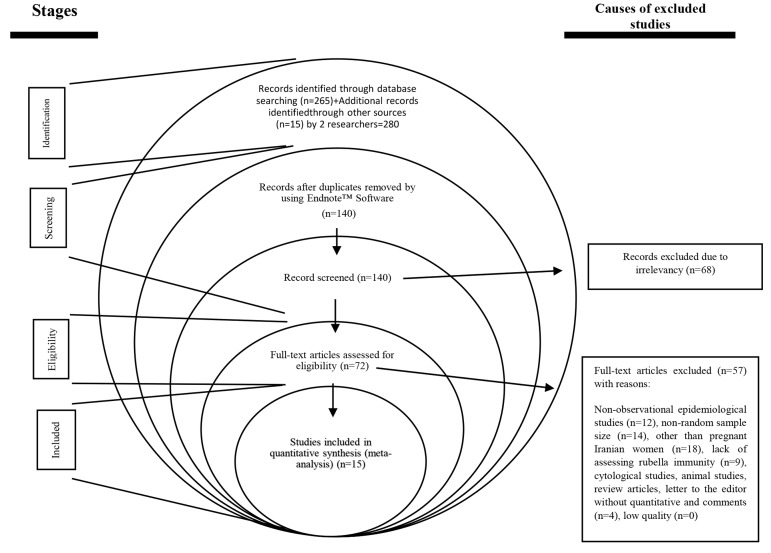
Study flow diagram.

In this systematic review, 280 articles were found by two researchers, among of which 264 articles were excluded because of the following reasons: duplicates (n=140), irrelevance (n=68), non-observational epidemiological studies (n=12), non-random sample (n=14), the sample size other than pregnant Iranian women (n=18); lack of assessing rubella immunity (n=9), and non-original studies (n=4, Fig.1). Finally, 15 studies comprising 7,601 pregnant woman with a mean age of 26.47 years [95% CI: 23.18-29.76] were entered into the meta-analysis process. The characteristics of each study are shown in Table 1.

**Table 1:** Summary of characteristics entered into the meta-analysis


First author, Published year	Year of study	Design	Place	Sample size	Age (mean ± SD)	Method	Immunity (%)	Quality	Ref.

Akbarian et al., 2007	2004	Cross-sectional	Tehran	810	21.9 ± 2.4	ELISA	85.5	High	(17)
Ghafourian Boroujerdnia et al., 2003	2000	Cross-sectional	Ahvaz	250	NR	HAI	92	High	(18)
Doraji et al., 2009	2009	Cross-sectional	Tehran	120	NR	ELISA	91.6	Medium	(19)
Pakzad and Moattari,1987	1986	Cross-sectional	Ahvaz	100	NR	HAI	90	Medium	(20)
Mokhtari et al., 2010	2007	Cross-sectional	Mashhad	73	26.7 ± 6.5	ELISA	90.4	High	(27)
Amini et al., 1996	2009	Cross-sectional	Tehran	210	NR	ELISA	94.3	Medium	(28)
Ashraf Ganjoei and Mohammadi, 2001	1997	Cross-sectional	Kerman	410	26.58 ± 5.5	ELISA	94.6	High	(29)
Pakzad and Ghafourian, 1995	2004	Cross-sectional	Dezfull	500	NR	HAI	74.8	Medium	(30)
Modarres, 2000	1996	Cross-sectional	Tehran	3008	NR	HAI	94	Medium	(31)
Bagheri Josheghani et al., 2015	1993	Cross-sectional	Kashan	80	30 ± 5.2	ELISA	92.5	High	(32)
Honarvar et al., 2013	2010	Cross-sectional	Shiraz	175	27.3 ± 5.3	ELISA	96	High	(33)
Ghafourian Boroujerdnia, 2001	2011	Cross-sectional	Ahvaz	300	NR	ELISA	78	High	(34)
Majlessi et al., 2008	1990	Cross-sectional	Tehran	965	NR	ELISA	91.1	High	(35)
Eslamian, 2000	2004	Cross-sectional	Tehran	500	NR	HAI	76	Medium	(36)
Ghaderi and Ghaderi, 2016	1995	Cross-sectional	Birjand	100	NR	ELISA	94	Medium	(37)


HAI; Haemagglutination-inhibition, ELISA; Enzyme-linked immunosorbent assay, NR: Not reported. and SD; Standard deviation.

### Pooled rubella immunity

**Fig 2:**
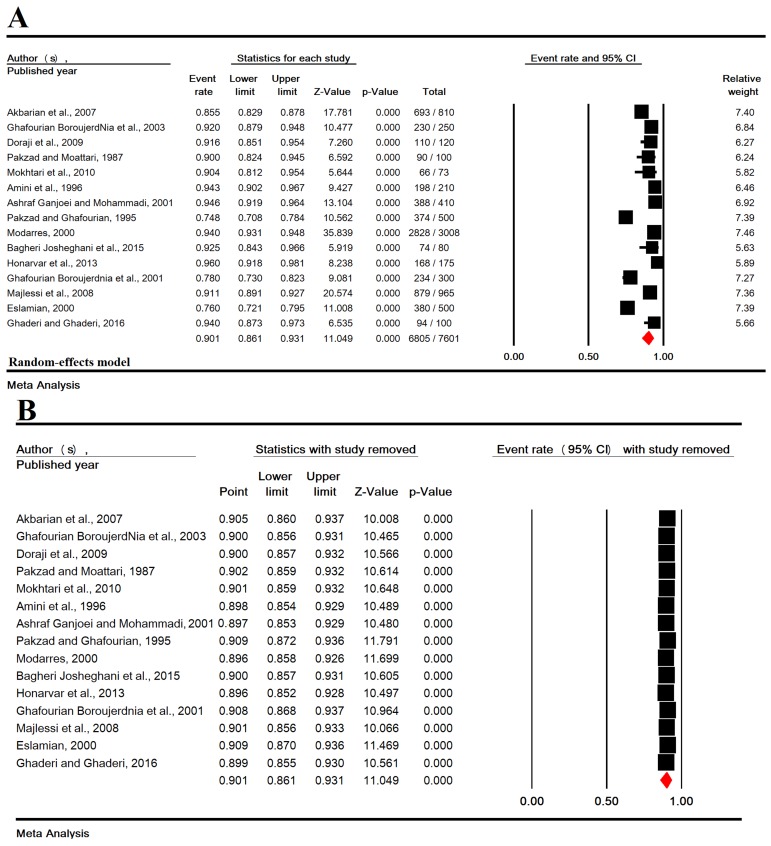
Forest plot for rubella immunity in pregnant Iranian women. **A.** Pooled estimate and **B.** sensitivity analysis.

The heterogeneity in this study was estimated to be high (P<0.001 and I2=95.7%). In an analysis of 7,601 pregnant women in Iran, rubella immunity rate was found to be 90.1% (95% CI: 86.1-93.1, Fig.2A). The lowest and highest rates were related to the studies in Dezful [74.8% (95% CI: 70.8-78.4)] (30) and Shiraz [96% (95% CI: 91.8-98.1)] (33), respectively. Forest plot for analysis of sensitivity was performed by removing one study at the same time to test the stability of the pooled. The results are shown in Figure 2B.

### Results of the subgroup analysis

Quantity of studies in the South, East and Central regions of Iran were 2, 9 and 4 studies, respectively. Rubella immunity rate for these regions was 93.3% (95% CI: 88.7-96.2), 87.8% (95% CI: 79.0-93.1) and 90.1% (95% CI: 85.4-93.4), respectively. This difference was not significant (P=0.286, Table 2). Sub- group analysis of rubella immunity rate based on quality of the studies was not significant (P=0.751, Table 2).

Rubella immunity rate, based on the ELISA method, was 91.4% (95% CI: 87.8-94.0) and based on the HIA method was 87.2% (95% CI: 74.3-94.1). Sub-group difference was not significant (P=0.355, Table 2). Sub-group analysis of rubella immunity rate based on national vaccination program is shown in Table 2. The difference was not significant (P=0.398). 

**Table 2:** Rubella immunity in pregnant Iranian women subgrouped using regions, quality of studies, diagnostic method and national vaccination program by random effects model


Variable		Study (n)	Sample size (n)	Heterogeneity				95% CI	Pooled estimate (%)
							Q			df	P value	I^2^ (%)		

Regions	Center	4	5793	188.24	8	<0.001	95.75	85.4-93.4	90.1
	South	2	483	1.87	1	0.171	46.65	88.7-96.2	93.3
	East	9	1325	57.04	4	<0.001	92.98	79.0-93.1	87.8
	Test for subgroup differences: Q=2.50, df(Q)=2, P=0.286								
Quality of the studies	High	8	3063	71.50	7	<0.001	90.21	86.5-93.6	90.7
	Medium	7	4538	251.62	6	<0.001	97.24	80.3-94.6	89.4
	Test for subgroup differences: Q=0.27, df(Q)=1, P=0.751								
Diagnostic method	ELISA	10	3243	88.14	9	<0.001	88.14	87.8-94.0	91.4
	HIA	5	4358	241.90	4	<0.001	98.34	74.3-94.1	87.2
	Test for subgroup differences: Q=0.85, df(Q)=1, P=0.355								
National vaccination program	Before	8	5278	291.49	7	<0.001	97.6	80.6-93.6	88.6
	After	7	2323	26.67	6	<0.001	79.34	88.1-93.9	91.5
	Test for subgroup differences: Q=0.71, df(Q)=1, P=0.398								


### Association of rubella immunity rate with age, occupation or accommodation place

**Fig 3:**
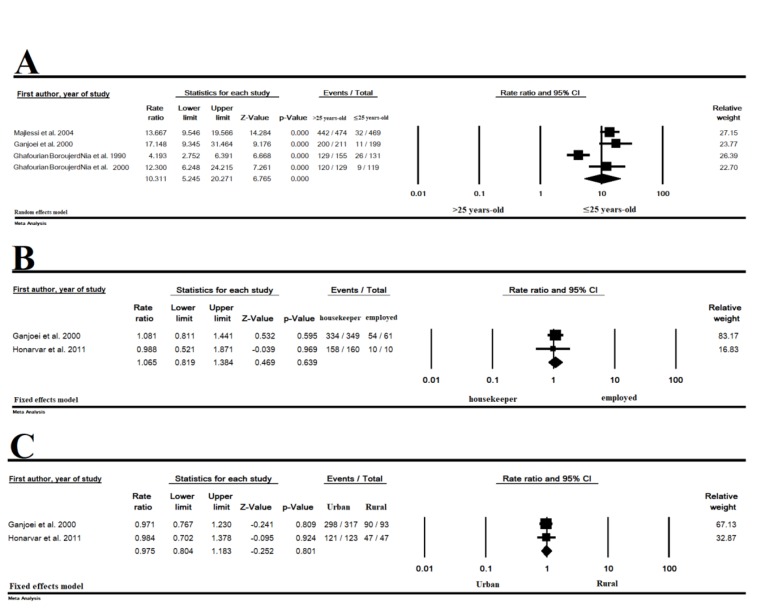
The association between rubella immunity rate and variables. **A.** Age, **B.** Occupation, and **C.** Place of residence.

The rubella immunity among pregnant women was significantly associated with age (.25 versus >25 years old) [OR=10.31 (95% CI: 5.24-20.27, P<0.001)], but it was not significantly associated with occupation (employed versus housekeeper) [OR=1.06 (95% CI: 0.81-1.38, P=0.639)] and place of residence (urban versus rural) [OR=0.97 (95% CI: 0.80-1.18, P=0.801)] (Fig.3).

### Meta-regression

**Fig 4:**
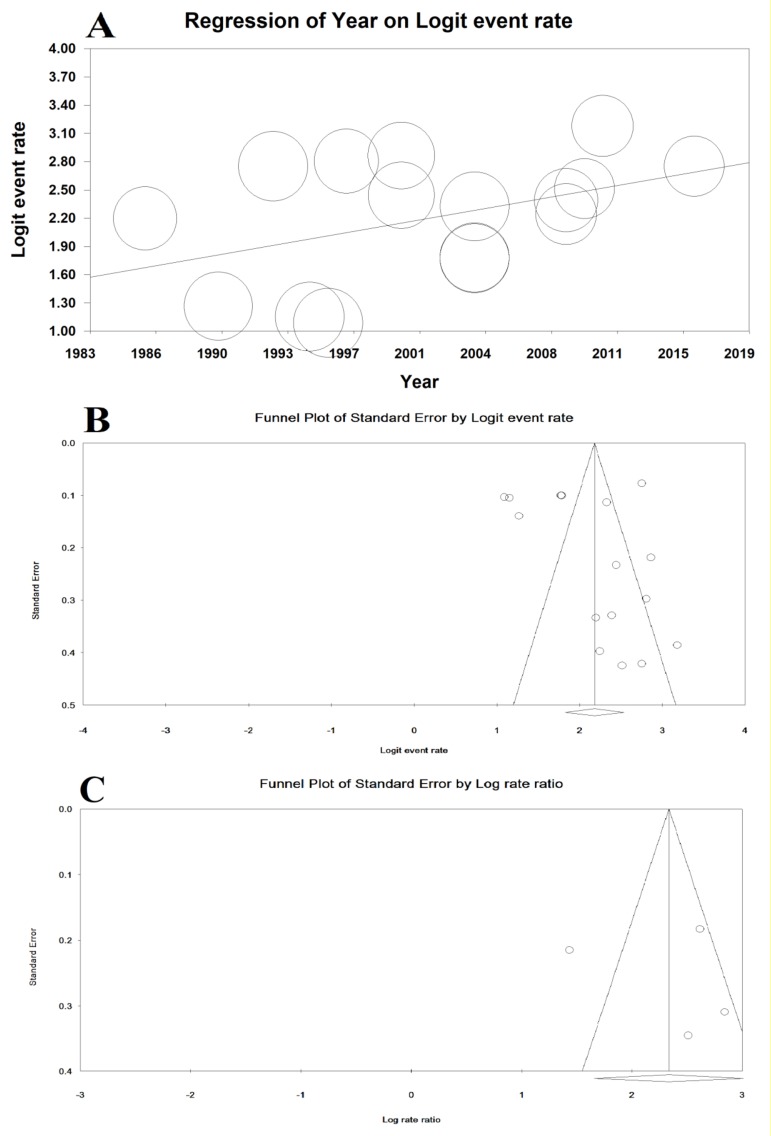
Meta-regression and publication bias. **A.** Meta-regression model of rubella immunity based on the year of the study, **B.** Publication bias for rubella immunity rate, and **C.** Relation between immunity and age.

Meta-regression of rubella immunity rate for the year of study was not statistically significant (P=0.164, Fig.4A).

### Publication bias 

In the evaluation of publication bias for rubella immunity rate, a funnel plot was drawn and P values based on Egger and Begg’s tests were estimated to be 0.45 and 0.75, respectively. In addition, rubella immunity rate were respectively 0.79 and 0.73 for association between rubella immunity rate and age, while they were not statistically significant (Fig.4B, C).

## Discussion

The results showed that rubella immunity rate among pregnant Iranian women was 90.1% and rubella immunity was not significantly associated with geographic regions, quality of the studies, diagnostic methods, vaccination program, occupation, place of residence, and year of the studies, occupation, place of residence, and year of the studies, but it was significantly associated with age.

CRS is declining in the world due to increased rubella vaccine coverage (38, 39). However, it remains a threat and a costly disease in some areas, where pregnant women were not immunized and protected against rubella virus. According to WHO, the primary objective of vaccination against rubella is the prevention of CRS. For this reason, immunization with rubella-containing vaccine is recommended for adult girls, women of childbearing age, or both (40).

In Iran, as a member of WHO, prevention and control of measles and rubella (MR) is an important priority (40). Several studies, conducted in various provinces of Iran, revealed that the immunity rate against rubella in women of reproductive age (15-45 years) is 69.9-97% (41, 42), and according to the present study, the immunity rate against rubella in pregnant Iranian women was found to be 86.1- 93.1%.

Rubella immunity rate among the pregnant women has been reported in European (74-98%), African (53-95%) and Asian (54-96%) countries (43-53). The probable cause of these different reports may be due to the universal coverage of vaccination against rubella and different diagnostic methods. However, in this study, this difference was not statistically significant.

In the past, MR were endemic in Iran and most of the people were infected until puberty. Therefore, most women acquired immunity against measles, rubella and mumps in their reproductive age. In 2002, the Ministry of Health and Medical Education in Iran established a comprehensive strategy for the elimination of MR. This strategy was launched with the aim of vaccinating 33,579,082 people, aged 5-25 years old, and 98% of the target population were vaccinated. This successful measure led to a decline in the incidence of MR to less than one case per million (41).

In this study, the rubella immunity rate in pregnant women before and after national vaccination program was estimated to be 88.6 and 90.4%, respectively. This difference was not statistically significant. It can be said that the high prevalence of IgG antibody seroprevalence during the years before implementation of vaccination programs is due to the high incidence of rubella and immunity through contact with the virus. In other countries such as Mexico, vaccination coverage was carried out from 1998 and this has been increased in Mexican pregnant women (14, 54).

In the present study, immunity rate against rubella was estimated 91.4% and 87.2%, using respectively ELISA and HAI methods. The specificity and sensitivity of the ELISA method for determining antibody against rubella was reported 61.7% and 95%, respectively (55). Shekarchi et al. (56) also mentioned that ELISA method is as accurate as HAI method and it would reliable, if purified antigens and carefully prepared reagents were used.

In this study, rubella immunity rate was higher in younger pregnant women. In a study performed by Alvarado- Esquivela in Mexico (14), age and socioeconomic level were significant and the other risk factors, such as residence, education level and occupation were not significant. In the study conducted by Hamdan et al. (48) in Sudan, the examined risk factors such as age, education level, gestational age, history of jaundice and body mass index were not significant. In another study in United States, international travel was demonstrated as a risk factor (55). Thus, it can be stated that each region has its own set of risk factors.

Limitations of the present study, including: i. The Iranian databases could not be used for advance search and ii. Many risk factors such as the year of birth and etc., were neglected.

## Conclusion

This meta-analysis provides information about rubella immunity in pregnant women. Although this study showed that the level of immunity in pregnant Iranian women is acceptable, it is recommended to perform anti-rubella antibody screening for all women of childbearing age.

## References

[1] Goodson JL, Masresha B, Dosseh A, Byabamazima C, Nshimirimana D, Cochi S (2011). Rubella epidemiology in Africa in the prevaccine era, 2002-2009. J Infect Dis.

[2] Lambert N, Strebel P, Orenstein W, Icenogle J, Poland GA (2015). Rubella. Lancet.

[3] Stock I (2012). Rubella (German measles)-still a major infectious disease. Med Monatsschr Pharm.

[4] Figueroa-Damián R, Ortiz-Ibarra FJ, Arredondo-García JL, Ahued-Ahued JR (1999). The outcome of pregnancies complicated by rubella, 1990-1997. Salud Publica Mex.

[5] Centers for Disease Control and Prevention (CDC) (2013). Nationwide rubella epidemic--Japan, 2013. MMWR Morb Mortal Wkly Rep.

[6] Saraswathy TS, Rozainanee MZ, Asshikin RN, Zainah S (2013). Congenital rubella syndrome: a review of laboratory data from 2002 to 2011. Southeast Asian J Trop Med Public Health.

[7] Dewan P, Gupta P (2012). Burden of Congenital Rubella Syndrome (CRS) in India: a systematic review. Indian Pediatr.

[8] Solórzano-Santos F, López-Kirwan A, Alvarez y Muñoz MT, Miranda-Novales MG, Gadea-Alvarez T, Vázquez-Rosales G (2001). Congenital rubella syndrome in infants treated at a pediatrics hospital. Gac Med Mex.

[9] Mandel G, Bennett J, Dolin R (2005). Principles and practice of infectious Disease.

[10] Kasper DL, Braunwald E, Hauser S, Longo D, Jameson JL, Fauci AS (2005). Harrison’s principle’s of internal Medicine.

[11] Goldman L, Ausiello D (2004). Cecil text book of medicine.

[12] World Health Organization (WHO) (2012). WHO-recommended surveillance standard of rubella and congenital rubella syndrome.

[13] McLean HQ, Fiebelkorn AP, Temte JL, Wallace GS (2013). Centers for Disease Control and Prevention.Prevention of measles, rubella, congenital rubella syndrome, and mumps, 2013: summary recommendations of the Advisory Committee on Immunization Practices (ACIP). MMWR Recomm Rep.

[14] Alvarado-Esquivel C, Hernandez-Tinoco J, Sanchez-Anguiano LF, Ramos-Nevarez A, Cerrillo-Soto SM, Salas-Pacheco JM (2016). Rubella immune status in pregnant women in a northern Mexican city. J Clin Med Res.

[15] Zen YH, Shih CT, Kung WJ, Lee CH, Lin CC (2017). Rubella immunity in pregnant native Taiwanese and immigrants from Asian countries. Am J Trop Med Hyg.

[16] Jonas A, Cardemil CV, Beukes A, Anderson R, Rota PA, Bankamp B (2016). Rubella immunity among pregnant women aged 15-44 years, Namibia, 2010. Int J Infect Dis.

[17] Akbarian A, Haghgo A, Hamkar R, Haj Abdulbaghi M, Esteghamati A, Nategh R (2007). Prevalence of congenital rubella syndrome after inadvertent MR vaccination of pregnant women in 2003. Razi Journal of Medical Sciences.

[18] Ghafourian Boroujerdnia M, Mohammad Jaffari R, Khodadady A (2003). Seroepidemiology of Rubella among pregnant women in Ahwaz, Iran. Jundishapur Scientific Medical Journal.

[19] Doraji M, Niakan M, Mostafavizadeh M, Moradi M, Esmaeli M (2012). Evaluation of IgG antibody titer against rubella virus in the first trimester pregnant women in selected centers of Tehran. Journal of Ardabil University of Medical Sciences.

[20] Pakzad P, Moattari A (1987). Serological study of the immunity to Rubella infection among the adult girls, pregnant and non-pregnant women, resident in Ahwaz. Jundishapur Scientific Medical Journal.

[21] Sayehmiri K, Tavan H, Sayehmire F, Mohamadi I (2015). Prevalence of Epilepsy in iran using meta-analysis and systematic review. ZUMSJ.

[22] Sayehmiri K, Tavan H (2015). Systematic review and meta-analysis methods prevalence of peptic ulcer in IRAN. Journal of Govaresh.

[23] Moher D, Liberati A, Tetzlaff J, Altman DG (2009). PRISMA Group.Preferred reporting items for systematic reviews and meta-analyses: the PRISMA statement. Ann Intern Med.

[24] Richardson WS, Wilson MC, Nishikawa J, Hayward RS (1995). The well-built clinical question: a key to evidence-based decisions. ACP J Club.

[25] Wells GA, Shea B, O’Connell D, Peterson J, Welch V, Losos M (2011). The Newcastle-Ottawa Scale (NOS) for assessing the quality of nonrandomized studies in meta-analyses. http: //wwwohrica/programs/clinical_epidemiology/oxfordasp..

[26] Ades AE, Lu G, Higgins JP (2005). The interpretation of random-effects meta-analysis in decision models. Med Decis Making.

[27] Mokhtari H, Babapor N, Mahdavian Naghash Zargar Sh (2010). The prevalence of anti-rubella antibody in pregnant women. Med Sci J Islamic Azad Univ Mashhad.

[28] Amini B (1996). A study of relationship between antibody of Rubella antivirus and number of pregnancy. J Zanjan Univ Med Sci.

[29] Ashraf Ganjoei T, Mohammadi MM (2001). Determination of Rubella virus antibodies in pregnant women referring to Kerman Maternity Hospitals in 1999. J Reprod Infertil.

[30] Pakzad P, Ghafourian M (1995). On the immunity of pregnant women to Rubella and the congenital abnormalities caused by this virus in khuzestan. Sci Med J Ahwaz Jundishapur Univ Med Sci.

[31] Modarres Sh (2000). Rubella virus infection during pregnancy and the immunity level of pregnant women to Rubella virus.J Med Counc I.R. Iran.

[32] Bagheri Josheghani S, Moniri R, Baghbani Taheri F, Sadat S, Heidarzadeh Z (2015). Prevalence of serum antibodies to TORCH infection in the first trimester of the pregnancy in Kashan, Iran. Iranian Journal of Neonatology.

[33] Honarvar B, Moghadami M, Moattari A, Emami A, Odoomi N, Bagheri Lankarani K (2013). Seroprevalence of anti-rubella and anti-measles IgG antibodies in pregnant women in Shiraz, Southern Iran: outcomes of a nationwide measles-rubella mass vaccination campaign. PLoS One.

[34] Ghafourian Boroujerdnia M (2001). Increased immunity to rubella in pregnant women in Ahvaz city during 1989-99. Journal of Reproduction and Infertility.

[35] Majlessi F, Batebi A, Shariat M, Rahimi A, Azad TM (2008). Rubella serology in pregnant women attending health centres of Tehran University of Medical Sciences. East Mediterr Health J.

[36] Eslamian L (2000). Rubella seroprevalence in pregnant women in Shariatti Hospital, Tehran, Iran. Acta Medica Iranica.

[37] Ghaderi R, Ghaderi F (2016). Rubella immunity among pregnant women in Iran. MOJ Immunol.

[38] Metcalf CJ, Lessler J, Klepac P, Cutts F, Grenfell BT (2012). Impact of birth rate, seasonality and transmission rate on minimum levels of coverage needed for rubella vaccination. Epidemiol Infect.

[39] World Health Organization Rubella.

[40] Rubella vaccines (2011). WHO position paper. Wkly Epidemiol Rec.

[41] Esteghamati A, Gouya MM, Zahraei SM, Dadras MN, Rashidi A, Mahoney F (2007). Progress in measles and rubella elimination in Iran. Pediatr Infect Dis J.

[42] Mahmoodi M, Vahedi E (2007). Comparison of rubella immunity of Mashhad’s women before and after Measles-Rubella mass campaigns 2003-2005. Medical Journal of Mashhad University of Medical Sciences.

[43] Calimeri S, Capua A, La Fauci V, Squeri R, Grillo OC, Lo Giudice D (2012). Prevalence of serum anti-rubella virus antibodies among pregnant women in southern Italy. Int J Gynaecol Obstet.

[45] Uyar Y, Balci A, Akcali A, Cabar C (2008). Prevalence of rubella and cytomegalovirus antibodies among pregnant women in northern Turkey. New Microbiol.

[46] Onakewhor JU, Chiwuzie J (2011). Seroprevalence survey of rubella infection in pregnancy at the University of Benin Teaching Hospital, Benin City, Nigeria. Niger J Clin Pract.

[47] Tahita MC, Hübschen JM, Tarnagda Z, Ernest D, Charpentier E, Kremer JR (2013). Rubella seroprevalence among pregnant women in Burkina Faso. BMC Infect Dis.

[48] Hamdan HZ, Abdelbagi IE, Nasser NM, Adam I (2011). Seroprevalence of cytomegalovirus and rubella among pregnant women in western Sudan. Virol J.

[49] Fokunang CN, Chia J, Ndumbe P, Mbu P, Atashili J (2010). Clinical studies on seroprevalence of rubella virus in pregnant women of Cameroon regions. African Journal of Clinical and Experimental Microbiology.

[50] Ali S, Khan FA, Mian AA, Afzal MS (2014). Seroprevalence of cytomegalovirus, herpes simplex virus and rubella virus among pregnant women in KPK province of Pakistan. J Infect Dev Ctries.

[51] Lin CC, Yang CY, Shih CT, Chen BH, Huang YL (2010). Rubella seroepidemiology and catch-up immunization among pregnant women in Taiwan: comparison between women born in Taiwan and immigrants from six countries in Asia. Am J Trop Med Hyg.

[52] Al-Marzoqi AHM, Kadhim RA, Al-Janabi DKF, Hussein HJ, Al Taee ZM (2012). Seroprevalence study of IgG and IgM antibodies to toxoplasma, rubella, cytomegalovirus, Chlamydia trachomatis and Herpes simplex II in Pregnancy women in Babylon Province. Journal of Biology, Agriculture and Healthcare.

[53] Jubaida N, Mondal M, Kawsar N (2011). Seroprevalence of rubella antibodies in pregnant women. Journal of Armed Forces Medical College, Bangladesh.

[54] Macías-Hernández AE, Ponce de León S, Muñoz-Barrett JM, López-Jiménez F, Cano-Castro A, Vera-Peña A (1993). The seroepidemiology of rubella in a female population of reproductive age in Leon, Guanajuato. Salud Publica Mex.

[55] Wittenburg RA, Roberts MA, Elliott LB, Little LM (1985). Comparative evaluation of commercial rubella virus antibody kits. J Clin Microbiol.

[56] Shekarchi IC, Sever JL, Tzan N, Ley A, Ward LC, Madden D (1981). Comparison of hemagglutination inhibition test and enzyme-linked immunosorbent assay for determining antibody to rubella virus. J Clin Microbiol.

